# Epstein-Barr Virus Related Lymphoproliferations After Stem Cell Transplantation

**DOI:** 10.4084/MJHID.2009.019

**Published:** 2009-12-14

**Authors:** Simona Sica, Elisabetta Metafuni, Silvia Bellesi, Patrizia Chiusolo

**Affiliations:** Istituto di Ematologia, Università Cattolica Sacro Cuore, Divisione di Ematologia, Policlinico Universitario Agostino Gemelli

## Abstract

Epstein-Barr virus related lymphoproliferative disorders are a rare but potentially fatal complication of allogeneic stem cell transplantation with an incidence of 1–3% and occurring within 6 months after transplantation. The most relevant risk factors include the use of in vivo T-cell depletion with antithymocyte globulin, HLA disparities between donor and recipient, donor type, splenectomy etc. The higher the numbers of risk factors the higher the risk of developing Epstein-Barr virus related lymphoproliferative disorders. Monitoring EBV viremia after transplantation is of value and it should be applied to high risk patients since it allows pre-emptive therapy initiation at specified threshold values and early treatment. This strategy might reduce mortality which was >80% prior to the implementation of anti-EBV therapy. Treatment of EBV-LPD after allogeneic SCT may consist of anti-B-cell therapy (rituximab), adoptive T-cell immunotherapy or both. Rituximab treatment should be considered the first treatment option, preferably guided by intensive monitoring of EBV DNA while reduction of immunosuppression should be carefully evaluated for the risk of graft versus host disease.

## Introduction:

EBV is an ubiquitous lympho- and epitheliotropic gamma1-herpesvirus. Primary infection of Epstein-Barr virus (EBV) is transmitted by saliva and occurs in childhood in asymptomatic manner. EBV actively replicates in the epithelial cells of the oropharynx and it can subsequently infects recirculating B lymphocytes. In 50% of adolescents primary EBV infection may lead to acute infectious mononucleosis. This symptomatic condition is the consequence of the powerful antiviral T cell response to the EBV-driven B-cell proliferation^1^. Immuno-compromised patients present a lack of T-cell control that favours the polyclonal expansion of B-cell clones that are infected and immortalized. These cells may also acquire additional genetic lesions leading to oligoclonality and, eventually, to monoclonality of the B-cell proliferation^2^. The immunocompromised status of patients after hemopoietic stem cell transplantation (SCT) or solid organ transplantation (SOT) may destroy the normal balance between latently infected B cell proliferation and the EBV-specific T cell response. Hence, the increased number of latently infected B cells may lead to post-transplant lympho-proliferatives disorders (PTLD) that may have a nodal or extranodal localization into one specific site or may involve the allograft after SOT^3^.

EBV-related clonal lesions range from policlonal plasmacytic hyperplasia to the atypical monoclonal polymorphic B-cell hyperplasia until the onset of an aggressive non Hodgkin’s lymphoma. The majority of PTLD cases are EBV-positive and many show a latency III pattern of gene expression. In healthy individuals the outgrowth of EBV transformed B-cells is prevented by a cell mediated response realized by cytotoxic T-lymphocytes and MHC-unrestricted NK cells. The most important control against the proliferation of viral infected B cells involves CD8 + specific cytotoxic T cells (CTL), which recognize viral epitopes presented by MHC Class I molecules on the surface of infected B cell^4^.

## Definitions:

The guidelines on the management of EBV infection in patients with hematological malignancies and after SCT from the Second European Conference on Infections in Leukemia have been published recently^5^ and the following definitions related to diagnosis of EBV infection in the HSCT setting have been introduced: *EBV-DNA-emia*– detection of EBV-DNA in the blood. *Primary EBV infection*– EBV detected in a previously EBV-seronegative patient. *Probable EBV disease*– significant lympho-adenopathy (or other end-organ disease) with high EBV-DNA load in the blood, in the absence of other etiologic factors or established diseases. *Proven EBV disease* (*PTLD or other end-organ disease*) – EBV detected from an organ by biopsy or other invasive procedures with a test with appropriate sensitivity and specificity together with symptoms and/or signs from the affected organ *Pre-emptive therapy* in this setting is defined as any agent or specific cells given to an asymptomatic patient with EBV detected by a screening assay, while treatment of EBV disease is with agents or other therapeutic methods applied to a patient with EBV disease (proven or probable).

## PTLD after SCT: origin, risk factors and diagnosis:

The PTLD is a severe complication of prolonged immunosuppression. The first cases of these disorders have been described in the contest of immunosuppression secondary to SOT. Prior to the introduction of cyclosporine A, the occurrence of PTLD was extremely rare. The rate of PTLD has been significantly increased after the introduction of the triple therapy with cyclosporine, OKT3 antibody and antithymocyte globulin (ATG) but on the other hand the introduction of effective immunosuppression has undoubtedly changed the rate of success of SOT through a significant prolongation of survival of patients. In this setting the incidence has been reported up to 15%. EBV related PTLD following SOT develops, in most of the cases, in the first years post transplant^6^. Risk factors for developing this complication are the degree and duration of immunosuppression and the onset of the primary infection after transplant in EBV naïve recipients. A correlation between the type of transplanted organ and the occurrence of PTLD has been extensively reported in the literature. Thus, the different incidences of PTLD in solid organ transplantation are as following:1–3% in kidney and liver transplant, 1–6% in cardiac transplant, 2–6% in combined heart-lung transplant, 4–10% in lung transplant, and up to 20% in small intestine transplant^6^.

The majority of cases of PLTD in solid organ transplantation are of recipient origin although recently the donor origin has been consistently reported after SOT^7^.

The genetic origin of PTLD has been determined by means of different technique including microsatellite analysis, performed on DNA extracted from tumor cells, from donor biopsy specimen obtained form transplanted organ and from recipient DNA.

After allogeneic SCT the overall frequencies of PLTD is about 1%. In allogeneic stem cell transplantation EBV-PTLD is nearly always of donor origin. Reddiconto et al.^8^ have described a case of PTLD in a patient with secondary –MDS, receiving double cord blood stem cell transplantation. The analysis of chimerism on patient biopsy specimens, documented that 95% of malignant cells were of donor origin, in particular, of female cord blood unit engrafted. However, rarely PTLD may origin from recipient B cells. In a case-report of PTLD^2^ in a child with acute T cell leukemia it was established the recipient origin of this neoplasm. The EBV- seropositive patient received graft from an unrelated seropositive donor. An explanation of this peculiar situation is the possible selection of clonal EBV-infected B lymphocytes before allogeneic transplantation.

The major risk factors for EBV-PTLD following allogeneic stem cell transplantation are: T-cell depletion of the graft, the use of serotherapy with ATG or Campath for prophylaxis and treatment of graft versus host disease (GVHD), transplantation from an unrelated or haploidentical donor, cord blood as source of stem cells in a reduced intensity conditioning (RIC) regimen setting, HLA disparity donor-recipient, duration of immunosuppressive prophylaxis and, finally, the type of conditioning regimen.

Van Esser et al.^9^ showed that the probabilities of developing EBV reactivation, defined as detection of EBV viremia without symptoms, were high after both unmanipulated and TCD-allogeneic SCT and were 31% and 65%, respectively. Patients receiving a T-cell depleted SCT have a significantly higher risk for recurrent EBV reactivations. In this study, the main difference between unmanipulated and TCD graft was the subsequent development of PTLD, this malignancy occurring only after TCD-SCT. This may be due to an impaired ability of these patients to mount an effective immune response to the reactivating virus. The few number of EBV-specific memory T cells in T-cell depleted graft may play a major role in this condition. In particular, the combination of T-cell depletion and use of ATG mostly favours PTLD development, with respect to only T-cell depletion.

A number of studies suggest that T cell reconstitution after RIC-regimens may be delayed compared to conventional myeloablative regimens^10^. In fact, RIC regimens determine a profound immunosuppression rather than myeloablation, so, the subsequent profound immunosuppression and prolonged lymphopenia result in an increased incidence of viral reactivations and possibly PTLD development. The experience of Great Ormond Street Hospital^11^ reported extensive monitoring for EBV reactivation and PTLD development in 128 paediatric patients undergoing an allogeneic SCT. In this cohort, EBV viremia and PTLD were significantly more frequent after RIC than conventional SCT. In the first group of patients receiving RIC, 35% of patients (23/65) developed EBV viremia. Eight of these remained asymptomatic, five patients developed symptomatic viremia without PTLD and ten patients developed PTLD. In contrast, only 8.8% (6/68) of the patients receiving conventional SCT developed EBV viremia. All of them remained asymptomatic and none progressed to PTLD^12^. Juvonen E. et al., reported 19 cases of PTLD in a cohort of 257 patients undergoing allogeneic SCT of non T-depleted grafts from HLA-unrelated and sibling donors. Of the 55 patients transplanted from an unrelated donor and treated with ATG as part of the conditioning, 15% (8/55) developed PTLD. Treatment of active GVHD was able to favour PLTD occurrence. In fact, in the absence of acute steroid-resistant GVHD requiring with ATG, no patients receiving a graft from a sibling donor developed PTLD. In this study there was also a different incidence of PTLD with the use of different ATG commercially available^13^. In the review reported by Cohen et al., almost all reported cases of PTLD in RIC transplantation have received a combination of fludarabine and serotherapy or Campath. This combination is profoundly immunosuppressive and it induce an higher T-cell depletion in vivo. This was supported by the lower incidence of EBV reactivation observed after RIC cord blood transplantation (UCBT), without ATG as part of conditioning regimens^13^ although there are controversial data in this specific issue. Despite some reports showing a possible increase in the risk of EBV-PTLD after UCBT, the rate of PTLD appear to be comparable to those reported after HLA-matched unrelated myeloablative transplantation. Brustein et al. studied 335 UCBT after myeloablative or RIC conditioning regimen. They observed an overall rate of EBV related complication of 4.5%, in particular, 3.3% for myeloablative transplantations and 7% for non myeloablative transplantation. In the setting of non ablative transplantation, the use of ATG leads to an incidence of EBV related complications up to 20%, compared with 2% in the absence of ATG^14^.

A feature of PTLD following UCBT is a longer interval for malignancy development compared to peripheral blood or bone marrow SCT, in both myeloablative and non myeloablative transplantations. The absence of EBV specific memory T cell in cord blood may account for this behaviour.

In a group of patients receiving Campath in vivo, Chakrabarti^10^ observed an increasing overall incidence of infections and a significantly delayed T-cell recovery. Nevertheless, the incidence of EBV-PTLD was low with alemtuzumab: 1% in a cohort of RIC-SCT receiving alemtuzumab in vivo and 0% in stem cell recipients receiving alemtuzumab-treated grafts. This probably reflects the ability of this anti-CD52 antibody to decrease both B cells, the EBV reservoir, and T cell^15^. HLA-mismatch may have a role in the pathogenesis of PTLD because immune reconstitution is delayed after a mismatched graft and T-lymphocytes remain the most important system in EBV infection control.

In a multivariate analysis from Sundin et al.^16^ HLA mismatch, splenectomy, and EBV seronegativity of recipients were significant risk factors for PTLD development. The incidence of PLTD was 0.26% among patients without risk factors. Patients with one risk factor had a probability of developing PTLD of 8.2% and those with two risk factors a probability of 35.7%. Interestingly all patients developing PTLD received an anti T-cell prophylaxis with ATG.

## EBV-DNA monitoring: predictivity of PTLD development and role in pre-emptive therapy:

As PTLD may evolve from a polyclonal disorder to a more aggressive monoclonal variant, an early diagnosis is relevant. In the last years, PCR monitoring of EBV viral load, a minimally invasive technique, has been increasing for the prevention and early detection of post transplant lymphoproliferative disease with the aim of export the successful paradigm of CMV pre-emptive strategy after SCT. EBV viremia was initially carried out by using qualitative PCR analysis and more recently by quantitative PCR technique.

The observed EBV loads in healthy donors are very similar in all the studies and are typically very low or undetectable. This depends on the low frequency of EBV positive B cells in the circulation that are lesser than 10 per 10^6^ in healthy carriers. In transplant recipients without EBV related diseases, the circulating viral load is generally higher than those reported in healthy donors, probably as a consequence of the shift of the immune balance towards the virus.

Even if the mean EBV load in the peripheral blood seems to be higher in HSCT recipients with PTLD, than in recipient without PTLD, patients with PTLD may present a broad range of PCR value within 1–5 logs. In addition, these values often overlap with the results of patients not developing PTLD. Thus we are not able to detect on the basis of viral EBV load patients progressing to EBV-PTLD. As a result pre-emptive treatment is now used in patients with increasing viral load or over the threshold of 10^3^ copies/ml or 200 copies per 10^5^ PBMC, empirically in many transplant centers with the aim to reduce the progression rate to PTLD.

Another obstacle for the interpretation of EBV data after SCT is related to different PCR monitoring analysis in published studies and many data are based on retrospective analysis of single center experience. In addition there is no general consensus on which is the best specimen to adopt (plasma, peripheral blood mononuclear cells, whole blood). In fact the determination of cell-associated EBV DNA loads could be interpreted as a marker of EBV-induced cell proliferation while plasma levels of EBV DNA could be expression of either virus production or the release of episomal DNA from apoptotic cells, or both^17^. A general consensus has been obtained on what should be the ideal interval of serial monitoring and this is based on the assumption that the doubling time of EBV viral load can be as short as 46–56 h. Thus, PCR monitoring should be performed once a week, at least, in order to optimized the detection of an elevated viral load before the clinical presentation of malignancy.

The sensitivity of EBV-DNA for the diagnosis of PTLD ranges from 78% and 100% in symptomatic patients, but is only between 50% and 80% when used for preemptive diagnosis. Subclinical reactivation of EBV detectable by PCR occurs from 20% to 60% of HSCT recipients without any PTLD symptoms and the positive predictive role of PCR monitoring for EBV remains highly variable^18^. EBV viremia by itself does not seems to predict PTLD development. EBV DNA levels are most suitable to confirm diagnoses of EBV PTLD, thereby permitting early and effective interventions.

## Clinical symptoms of EBV-related PTLD:

Manifestations of post-transplant EBV infection, both primary and reactivation, include EBV-associated enteritis with multiple ulcers, EBV-related hepatitis, encephalitis, and fulminant EBV-associated hemophagocytosis. These complications are, however, infrequent. Chronic active EBV infection characterized by chronic or recurrent infectious mononucleosis-like symptoms such as fever, hepatosplenomegaly, persistent hepatitis, and extensive lymphadenopathy has also been described. The most severe manifestation of EBV infections following allogeneic SCT^4^. is EBV-associated PTLD. EBV-associated tumors (reactivation syndromes) include lympho-proliferative disease (LPD), Burkitt’s lymphoma/non-Hodgkin lymphoma (NHL), nasopharyngeal carcinoma, natural killer (NK)-cell leukemia, Hodgkin’s disease, hemophagocytic lymphohistiocytosis and angioblastic T-cell lymphoma. Histopatological classification has been recently updated by the World Health Organization (WHO)^19^.

## Diagnosis of EBV disease:

Diagnosis of LPD or PTLD must be based on symptoms and/or signs consistent with lymphoproliferative process developing after SCT, together with detection of EBV by an appropriate method applied to a specimen from the involved tissue. Definitive diagnosis of EBV-PTLD requires biopsy and histological examination (including immunohistochemistry or flow cytometry for CD19+ and CD20+). It is important to remember that CD20 can be downregulated on lymphoma cells after therapy. EBV detection in biopsy specimen requires detection of viral antigens or *in situ* hybridization for the EBER (Epstein–Barr-encoded RNA) transcripts.

Definitive diagnosis of LPD or PTLD must be based on symptoms and/or signs consistent with PTLD together with detection of EBV by an appropriate method applied to a specimen from the involved tissue.

## Treatment strategies for EBV-related PTLD:

The mortality from PTLD after HSCT was >80% prior the implementation of anti-EBV therapy. Treatment strategies for EBV-related PTLD include reduction of immunosuppression, anti-B cell monoclonal antibodies, conventional chemotherapy and radiation. No effect of antivirals drugs can be expected with respect to prevention and treatment of EBV-LPD. When PTLD is established, standard lymphoma chemotherapy is used in combination with rituximab^4^. A recent approach includes the infusions of donor-derived lymphocytes in SCT patients with PTLD, or infusions of HLA-matched EBV-specific cytotoxic T lymphocytes (CTLs) in PTLD both after SOT and SCT.

The reduction of immunosuppression therapy is usually the first-line approach for PTLD transplantation but this may not be feasible in patients with active GVHD.

The overall success rates of EBV-related PTLD were lower than after pre-emptive therapy, reaching 63% and 88.2% of total EBV-DNA clearance with rituximab and CTL therapy, respectively. Thus, because of the progressive nature of PTLD, an early or pre-emptive treatment with either anti-B cell monoclonal antibodies or donor-derived EBV-specific cytotoxic-T lymphocytes is appealing^20^. Pre-emptive strategy is now empirically used in many transplant centers and rituximab is the treatment of choice although there are no consistent data on the best dosage, number of doses and interval between doses of rituximab.

Wagner et al.^21^, studied a cohort of 111 patients who underwent myeloablative HSCT from HLA-matched unrelated donor or HLA-mismatched sibling donor, all receiving ATG or Campath and TCD-graft. EBV viral load from of whole blood was monitored. No patients with consistently low EBV DNA levels developed PTLD. Sixteen patients presented EBV-DNA levels exceeding 4000 copies/μg PBMC DNA in two or more occasions and nine patients had a single occurrence of high EBV load.

Among them, eight developed symptoms of PTLD. Hence, the detection of EBV-DNA levels > 4000 copies /μg PBMC DNA had a sensitivity of 100% for the prediction of early PTLD but a specificity of only 50% (8/16). All patients received treatment: two patients received CTLs, five patients received rituximab (1–4 dose of 375 mg/m2/die) and another one received CTLs and rituximab. Clinical symptoms associated with PTLD disappeared and EBV load decreased to a normal value in 7 out of 8 patients.

In another study, Van Esser et al. employed PCR for an early diagnosis and pre-emptive treatment of the EBV related PTLD. The threshold of 1000 copies of viral DNA genome per millilitre was set as the reference level for a high predictive value of PTLD development. They studied 49 patients receiving a partial T-cell depleted transplant from HLA-matched sibling donor or matched unrelated donor. EBV reactivation with a viral load above 1000 copies/ml was detected in 17 out of 49 patients (35%). Among them, 2 patients presented an active PTLD and received two infusions of rituximab, obtaining complete and persistent clearance of EBV viral load. The other 15 patients presented a subclinical reactivation and were treated with rituximab pre-emptively. Fourteen patients have shown a complete response after a single dose of rituximab. The only non responder patient showed an increased viral load and a progression to EBV related PTLD and was treated with a second dose of rituximab and DLI and achieved a clinical response. Thus, pre-emptive rituximab selectively administered to high-risk patients, abrogates EBV reactivation and reduces the incidence of EBV-PTLD^22^.

Annels et al.^23^, showed that EBV specific T cell reconstitution may be a second important parameter to guide pre-emptive treatment. The authors defined the EBV reactivation as an EBV viral load > 1000 copies at 2 consecutive time points. This was considered a condition of high risk to develop EBV-PTLD, together with a graft from an EBV-seropositive, unrelated or mismatched family donor and ATG administration. Eight out of fifty patients presented these features and were pre-emptive treated with a single standard dose of rituximab. T cell reconstitution was studied in all patients. Six out of 8 treated patients presented a significant T cell reconstitution and an EBV specific memory T cells expansion during EBV reactivation, with a contemporary decrease in EBV DNA load. Additional evidence for the antiviral potential of this T cell reconstitution was prospectively confirmed by a cohort of 14 HSCT recipients at risk for EBV-LPD. Three out of 14 patients reactivated EBV. Two patients developed a significant and rapid T cell expansion during the beginning of viral reactivation and they obtained a clearing of viral load without rituximab administration. T cell recovery was absent only in one patient successfully treated with rituximab.

We have prospectively monitored EBV reactivation in a cohort of 104 patients receiving HSCT between February 2005 and August 2009. Seventy-nine patients received an allogeneic SCT and twenty-five patients received an autologous SCT. EBV reactivation rate was of 16% (4/25) with a median time to the reactivation of 115 days in the autologous SCT group. In allogeneic transplant group EBV reactivation was of 60.76% (48/79) with a median time to reactivation of 59 days (range 3–840), and a median value of viral genome copies per ml of 1.577.017 (range 408–60.000.000) (Table 1). Strikingly the incidence od EBV reactivation varied across the different stem cell source being more frequently after bone marrow (100%) or peripheral blood (64.2%) transplant compared to CB graft (12.5%). In patients receiving bone marrow transplant there was an earlier viral reactivation compared to patients receiving peripheral or CB transplant. Due to the small number of patients receiving bone marrow these data should be interpreted with caution. Type of donor also affected the incidence and time of reactivation. The incidence was 86.3% using unrelated donors and 57.1% using sibling transplant (p= 0.016). In patients receiving ATG or Campath as part of GVHD prophylaxis, the rate of reactivation was 93.3%. Also some patients presented two or more subsequent viral reactivations. The major conditions affecting multiple reactivations were underlying lympho-proliferative diseases (65.2%), myeloproliferative diseases (36%) at transplant and the occurrence of GVHD (64%) (Table 2).

In fourthy-eight patients who reactivated EBV, serum protein electrophoresis was carried out. A γ-peak, indicating the presence of a monoclonal gammopathy (MG), was detected in 31 patients (64.5%). Twenty-seven patients with MG were further investigated with immunofixation on serum and urine. Monoclonal gammopathy was present in 22/27 patients and in 5 patients the Ig isotype was not identified (Table 3). Subtype was: a single IgG monoclonal gammopathy in 11 patients (IgGκ in 5 cases, IgGλ in 6 cases), a single IgM monoclonal gammopathy in two patients (IgMκ in one case and IgMλ in the other one), a single IgAκ monoclonal gammopathy in one patient, and a double monoclonal gammopathy in the others 8 patients (IgGκ+IgGλ in 3 cases, I IgGκ+IgMλ in 3 cases, gMλ+IgGλ in one case, and IgMκ+IgGκ in the last patient).

Six patients developed PTLD (12.5% of the patients with EBV reactivation and 7.6% of the allo-SCT recipients). In five of these, PTLD followed EBV DNA detection in peripheral blood, while in the last patient PTLD was diagnosed by biopsy, in the absence of previous EBV detection. All patients with PLTD presented a γ-peak at the serum protein electrophoresis. In five patients a monoclonal gammopathy was confirmed by immunofixation: four patients presented a single monoclonal gammopathy (IgGk in two cases, IgGλ in one case and IgAk in one case) and one patient presented a double monoclonal gammopathy IgMk+IgGλ (Table 4).

Treatment with rituximab (375mg/m2/die for 1–4 doses) was administered to all cases of PTLD and to others 12 asymptomatic or symptomatic patients with an high number of EBV genome copies in order to avoid the progression to PTLD. The vast majority of patients (88.9%) treated with rituximab showed a sharp viral load clearance. In 11 out of 18 patients treated with rituximab (61%) abnormalities of serum protein electrophoresis normalized after treatment. After rituximab, immunofixation was evaluated only in 10 out 18 treated patients. Five of the these patients showed the complete resolution of the MG after treatment. Two patients with PTLD died early after treatment: one patient from multi-organ failure and the other because of progressive disease. The remaining four patients presented resolution of symptoms and a drop in EBV viremia in peripheral blood until its disappearance. They are alive at follow-up with an overall survival of 6,12, 13 and 45 months, respectively.

Hence, the risk factors reported in the literature are substantially confirmed in this prospective study. Rituximab was effective for a prompt treatment of PTLD and useful as pre-emptive treatment limiting the progression of EBV reactivation to PTLD. Monoclonal gammopathy may help to define patients with EBV viremia at high risk of PTLD after SCT^24^. After SOT the presence of monoclonal gammopathy (MG) after organ transplantation has already been considered a risk factor for the development of PTLD^25^,^26^,^27^.

## Conclusions:

In summary, treatment options include manipulation of the balance between outgrowing EBV-infected B cells and the EBV CTLs response targeting the B cells with monoclonal antibody rituximab and conventional chemotherapy. The efficacy of this strategy is greater in early PTLD than in later disease. In the case of EBV-specific CTL it should be noted that this approach is not widely available. Rituximab is useful as a pre-emptive treatment in patients with high EBV viral load and with risk factors for PTLD (Table 5). Over-treatment is the rule and it has been calculated that at least 5.6 patients will be treated unnecessary in order to prevent 1 single case of EBV-PTLD^18^.

With current approaches the mortality from EBV-PTLD can be significantly reduced but preemptive treatment based on EBV viremia should be validate and diagnostic tests should be standardized in prospective trials. The search for unrecognized risk factors for EBV-related LPD after SCT is ongoing and includes biomarkers, immune-phenotype and genetic polymorphisms of both recipient and donor.

## Figures and Tables

**Table 1.** Characteristics of patients receiving allogeneic transplant.

Total of patients	79
Sex (M/F)	51/28
Median age (range)	44.6 (10–70)
Diagnosis	36 AML, 5 NHL, 1 RAEB-2, 8 CLL, 3 MF, 1 PL, 4 MM, 1 LL, 1 HD, 18 ALL,1 AA
Conditioning (Abl/RIC)	37/42
GvHD prophylaxis	22/79 ATG (27.8%) 5/79 (6.3%)Campath-1H
Donor (sib/MUD)	29/50
Stem cell source	CB 8/79 (10.1%), PB 67 /79 (84.8%), BM 4/79 (5.1%)
GvHD (acute vs chronic)	27 vs 24

AML: acute myeloid leukemia; NHL: non-Hodgkin’s lymphoma; CLL: chronic lymphocitic leukemia; MF: myelofibrosis; PL: plasmacell leukemia; LL: lymphoblastic lymphoma; HL: Hodgkin’s disease; ALL: acute lymphoblastic leukemia; MM: multiple myeloma; AA: aplastic anaemia; RAEB: refractory anemia with excess blasts Abl: ablative; RIC: reduced intensity conditioning.; MUD: matched unrelated donor; CB: cord blood, PB: peripheral blood; BM. bone marrow

**Table 2.** Characteristics of patients with EBV reactivation/PTLD after allogeneic SCT

Patients with EBV reactivation	48/79(60.7%)
Median time of reactivation (days-range)	55 (3–840)
Median EBV DNA copies/ml (range)	1.577.017,83 (408–60.000.000)
Number of reactivations	one 24/48 (50%) two 17/48 (35.4%) three 7/48 (14.6%)
PTLD	5/48 (10.4%)
Patients with peak in □-region	31/48 (64.6%)
Patients with Monoclonal Gammopathy	22/48 (45.8%)
Donor (sib/MUD)	28/20
Stem Cell Source	PB 43, CB 1, BM 4
Conditioning Regimen (abl/RIC)	21/27
ATG/Campath-1H	11/5
GvHD (acute vs chronic)	18 vs 7

**Table 3.** Monoclonal Gammopaties in EBV reactivating patients

EBV reactivation	48 pts
Presence of γ-peak	31 pts
Presence of monoclonal gammopathy	22
IgG	11 5 IgGk 6 IgGλ
IgM	2 1 IgMk 1 IgMλ
IgAk	1
IgGk+IgGλ	3
IgMλ+IgGλ	1
IgGK+IgMλ	3
IgMk+IgGk	1

**Table 4.** Characteristics of patients with PTLD

Patients with PTLD	6

Patients with EBV + PCR	5/6 (83.3%)

Patients with histological diagnosis of PTLD	2/6 (33.3%)

Donor	6 MUD

Stem Cell Source	5/6 (83.3%) PB 1/6 (16.6%) CB

Conditioning Regimen (abl/RIC)	4/2

GVHD prophylaxis	5/6 (83.3%) ATG 1/6 (16.6%) Campath-1H

Presence of □-peak	6/6

Presence of Monoclonal Gammopathy	5/6 (83.3%)
IgGk	2/5 (40%)
IgGλ	1/5 (20%)
IgAk	1/5 (20%)
IgMk+IgGλ	1/5 (20%)

GvHD (acute vs chronic)	2 vs 1

**Table 5.** Cumulative results of various therapeutic approaches in preemptive therapy and therapy of PTLD adapted from Styczynski et al^20^

**Therapeutic approach**	**Cumulative number of treated patients**	**Cumulative number of patients with cure or improvement**	**Comments**
rituximab in pre-emptive therapy	341	306 (89.7%)	Administration of rituximab was often combined with other therapeutic approaches.
rituximab in therapy of PTLD	146	92 (63%)	
CTL in pre-emptive therapy	135	127(94.1%)	In most cases reported as a single therapy
CTL in therapy of PTLD	17	15 (88.2%)	
reduction of immunosuppressive therapy	76	43 (56.6%)	In most cases others therapies were also used
Donor lymphocyte infusion	39	16(41.0%)	In most cases others therapies were also used
Chemotherapy	15	4(26.7%)	In most cases others therapies were also used
Cidofovir in combination with rituximab in pre-emptive therapy	42	37(92.5%)	In all cases others therapies were also used
Antiviral agents in therapy of PTLD	62	21(34.4%)	
